# DAF-YOLO: Detection of Unsafe Behaviors on Construction Sites

**DOI:** 10.3390/s25237216

**Published:** 2025-11-26

**Authors:** Qi Xu, Xiang Cheng, Xiaoxiong Zhou, Xuejun Jia, Xiaoxiao Wang, Zhihan Shi, Shanshan Huang, Guangming Zhang

**Affiliations:** 1 College of Electrical Engineering and Control Science, Nanjing Tech University, Nanjing 211816, China; xenon@njtech.edu.cn (Q.X.); chengxiang2024@njtech.edu.cn (X.C.); zhouxx@njtech.edu.cn (X.Z.); 2 China Construction Second Engineering Bureau Co., Ltd., Beijing 100160, China; lc-1206@njtech.edu.cn (X.J.); jitianyu@njtech.edu.cn (X.W.); 3 School of Electrical and Energy Engineering, Nantong Institute of Technology, Nantong 226001, China; 20256115@ntit.edu.cn (Z.S.); 20252126@ntit.edu.cn (S.H.)

**Keywords:** YOLO, unsafe behavior, object detection, detection model, construction site safety

## Abstract

Construction sites are complex environments, and unsafe behaviors by workers, such as not wearing safety helmets or reflective vests, can easily lead to accidents. When using target detection technology to detect unsafe behaviors, the results are often unsatisfactory due to the complexity of the background and the small size of the targets. This paper proposes an unsafe behavior detection algorithm based on dual adaptive feature fusion. The algorithm is based on YOLOv5, introducing a front-end adaptive feature fusion module (FE-AFFM) at the head of the backbone network for deep data processing, improving the model’s feature extraction capability in complex backgrounds. Simultaneously, a back-end adaptive feature fusion module (BE-AFFM) is introduced at the tail of the network to strengthen feature fusion. In the experimental verification phase, this paper selects a self-made laboratory dataset and verifies the effectiveness of the improved algorithm through ablation experiments, algorithm comparisons, and heatmap analysis. The average accuracy of the improved algorithm is 3.6% higher than the baseline model, and the detection effect on small targets is significantly improved, meeting the actual needs of construction sites. This paper also selects the publicly available dataset SHWD for algorithm comparison experiments. The results show that the improved algorithm still has a significant advantage over mainstream algorithms, verifying the generalization ability of the improved model.

## 1. Introduction

Safety in production has always been a top priority in the construction industry. Statistics show that in 2023, among 100,000 employees in China’s industrial, mining, and commercial enterprises, 1244 died in work-related accidents, a 4.2% increase from the previous year [1]. Most of these accidents stem from a lack of safety awareness and unsafe practices among production workers. In the construction industry, unsafe practices typically refer to actions that violate safety management regulations and may lead to personal injury or equipment damage. These mainly include not wearing a safety helmet as required, not wearing a reflective vest, and crossing warning lines. This article focuses on two categories of unsafe practices by construction workers: whether or not they wear safety helmets and whether or not they wear reflective vests. Supervising unsafe behaviors of construction workers can effectively prevent accidents. Traditional manual inspection methods are inefficient, subjective, and prone to missed detections, and are gradually being replaced by artificial intelligence technology. Currently, the mainstream approach combines monitoring equipment with machine vision, using object detection algorithms to identify unsafe behaviors on-site. Object detection algorithms mainly include traditional methods and deep learning-based methods. Traditional methods, represented by HOG [2] and SIFT [3], rely on manually designed features and struggle to handle object detection in complex backgrounds. Deep learning algorithms utilize convolutional neural networks to extract multi-layered and richer features, offering greater adaptability.

Deep learning object detection algorithms can be further divided into single-stage and two-stage algorithms. Two-stage algorithms, represented by R-CNN [4], first generate candidate boxes, then classify and regress them to determine the target’s location and category. Single-stage algorithms directly perform classification and regression operations, such as the YOLO (You Only Look Once) series [5,6,7,8,9,10,11,12,13] and SSD [14].

Unlike typical detection scenarios, construction sites contain a large amount of materials such as steel bars, formwork, and bricks. These materials are randomly piled up, and their colors and textures are complex and varied, easily leading to misjudgments by detection models and increasing the difficulty for detection algorithms to extract effective features. Furthermore, for the purpose of monitoring the entire site, cameras at construction sites are generally installed at high locations such as tower cranes, factory eaves, or poles. At high angles, the pixel area of the person being detected in the image is drastically reduced, particularly affecting the detection of small targets such as safety helmets. In summary, target detection technology at construction sites faces challenges such as complex environments and small target scales. To address this difficulty, this paper proposes an unsafe behavior detection algorithm based on dual adaptive feature fusion. This algorithm, based on YOLOv5s, enhances the network model’s ability to detect small targets by strengthening the beginning and end of the backbone network, meeting the actual needs of construction site scenarios.

The principal contributions of this paper are as follows:

1. An effective model for detecting unsafe behaviors at construction sites is proposed;
2. Introduction of a front-end adaptive feature fusion module at the head of the YOLOv5s backbone network, enhancing the model’s data mining capabilities at source;
3. The incorporation of a back-end adaptive feature fusion module at the tail end of the YOLOv5s backbone network enables more flexible feature extraction.

The remainder of this paper is organized as follows: Section 2 reviews the YOLO series of algorithms and related work on small object detection; Section 3 details the proposed method; Section 4 analyses the experimental results; Section 5 concludes the paper.

## 2. Related Work

In recent years, object detection technology has been the subject of extensive research, with the YOLO series of algorithms emerging as a focal point of investigation due to their efficiency and robustness. Furthermore, the detection of small objects has also attracted considerable attention as a prominent research direction. This section provides an overview of the relevant work, focusing on two key areas: the YOLO series algorithms and small object detection techniques.

### 2.1. Overview of YOLO Detection Algorithms

The YOLO family of algorithms represents one of the most influential developments in the field of object detection. First introduced by Joseph Redmon and colleagues in 2015, YOLO reframed object detection as a single regression problem, allowing a neural network to directly predict bounding boxes and class probabilities in an end-to-end manner for real-time performance.

YOLOv1 laid the groundwork for single-stage detection but struggled with small-object recognition and localization accuracy. YOLOv2 addressed these limitations by incorporating anchor boxes and batch normalization, which substantially improved detection stability and accuracy. Building upon this, YOLOv3 adopted a deeper Darknet-53 backbone and integrated the Feature Pyramid Network (FPN) structure to enhance multi-scale detection capability.

Since then, the YOLO framework has continued to evolve through successive versions up to YOLOv13.

This study chose YOLOv5 as the baseline model primarily for the following reasons:

1. After long-term development, the YOLOv5 framework is mature and its codebase is stable. Using newer versions of YOLO may lead to runtime failures due to environment incompatibility, affecting detection progress;
2. YOLOv5 has friendly resources and a complete toolchain, facilitating research and future industrial deployment [15,16,17].

**Figure 1:**
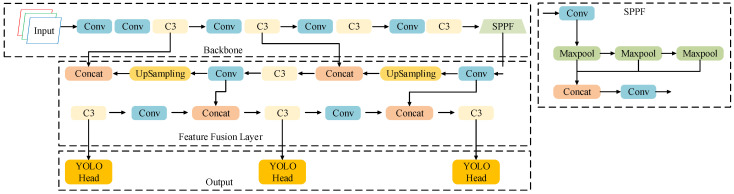
YOLOv5 Network Architecture Diagram.

Developed by the Ultralytics team in 2020, YOLOv5 consists of four main components: the input layer, backbone network, feature fusion layer, and output layer, as illustrated in Figure 1. The model is available in five variants—YOLOv5s, YOLOv5m, YOLOv5l, YOLOv5x, and YOLOv5n—which share the same architecture but differ in depth and width coefficients. Larger coefficients increase model complexity and parameter count, generally leading to nonlinear gains in detection accuracy [18].

In the YOLOv5 framework, object detection is achieved through a combination of convolution, pooling, and feature fusion operations. After preprocessing, input images are resized to 640 × 640 × 3, and features are extracted by the backbone network. The feature fusion layer then combines multi-scale feature maps to identify objects of different sizes, with final predictions produced by the detection head.

### 2.2. Advances in Small Object Detection Algorithms

Small object detection remains a classic challenge in computer vision. Due to minuscule dimensions and sparse feature information, such objects are easily overlooked or misclassified during detection, limiting the performance ceiling of many models. In recent years, numerous scholars have persistently pursued research in this domain. ZHANG [19] et al. enhanced the representation of weak features by introducing feature enhancement, fusion, and spatial context-aware modules to the YOLO algorithm. Su [20] et al. enhanced detection capabilities by adding a small object detection layer to YOLOv7, incorporating a spatially adaptive feature fusion detection head and deformable convolutions. Qi [21] et al. expanded the receptive field by modifying SPPF into the SPPF-S module within YOLOv8n, thereby preserving more features. Liu Zhaolong [22] et al. enhanced YOLOv7 by incorporating a small object detection layer, introducing the parameter-free attention mechanism SimAM [23] to achieve more accurate detection results. Currently, research cases addressing small object detection in construction site scenarios remain scarce.

## 3. Method

### 3.1. Front-End Adaptive Feature Fusion Module

The FE-AFFM consists of n Adaptive Feature Fusion Modules (AFFMs) and two convolutional layers. At the initial stage, a 1 × 1 convolution is applied to compress the channel dimensions of the input features. This operation reduces computational cost while making the feature representation more compact and expressive. The compressed features are then divided into two streams: one is transmitted directly, while the other is fed into multiple AFFMs to perform multi-scale extraction and deep feature enhancement. Afterward, the two feature branches are concatenated along the channel axis, allowing complementary information to be integrated and reorganized. A final 1 × 1 convolution further refines the fused representation by aggregating channel information, producing the module’s output.

**Figure 2:**
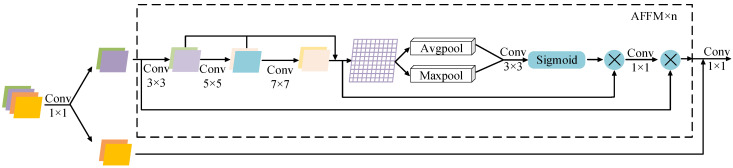
FE-AFFM.

The architecture of the FE-AFFM is shown in Figure 2. Each AFFM submodule is designed to perform deep feature mining. Upon entering an AFFM unit, the input feature is split into two parts. The first branch passes sequentially through three convolutional layers with kernel sizes of 3, 5, and 7, forming a residual connection that helps prevent gradient vanishing and enhances the network’s nonlinear representational capacity. The processed output then undergoes both average pooling and max pooling. The resulting feature maps are concatenated, convolved, and passed through a Sigmoid activation to normalize the response. A subsequent 1×1 convolution reduces the number of channels to ease the computational load of the following stages. The second branch from the module input is then fused with the deeply processed feature maps to generate the final output.

The AFFM submodule is repeated n times according to detection requirements; in this study, n is set to 3.

The mathematical formula for the FE-AFFM is as follows:

(1) $$F_{\mathrm{Split}}\left(X_{1},X_{2}\right)=F_{\mathrm{Split}}\left(\mathrm{Conv}\left(X\right)\right)$$

(2) $$X_{3}=\mathrm{Concat}\left({\mathrm{Conv}}_{3\times 3}\left(X_{1}\right),{\mathrm{Conv}}_{5\times 5}\left({\mathrm{Conv}}_{3\times 3}\left(X_{1}\right)\right),{\mathrm{Conv}}_{7\times 7}\left({\mathrm{Conv}}_{5\times 5}\left({\mathrm{Conv}}_{3\times 3}\left(X_{1}\right)\right)\right)\right)$$

(3) $$F_{\mathrm{AFFM}}=X_{1}⊙\mathrm{Conv}\left(X_{3}⊙\;\sigma \left({\mathrm{Conv}}_{3\times 3}\left(\mathrm{Concat}\left(\mathrm{AvgPool}\left(X_{3}\right),\mathrm{MaxPool}\left(X_{3}\right)\right)\right)\right)\right)$$

(4) $$F_{\mathrm{FE}\text{-}\mathrm{AFFM}}={\mathrm{Conv}}_{1\times 1}\left(\mathrm{Concat}\left(F_{\mathrm{AFFM}}^{3}\left(X_{1}\right),X_{2}\right)\right)$$

where *X* denotes the input data, and $X_{1}$, $X_{2}$ and $X_{3}$ represent intermediate variables.

### 3.2. Back-End Adaptive Feature Fusion Module

The structure of the SPPF module is illustrated in Figure 1. Positioned at the deepest layer of the backbone network, it employs three pooling operations to extract rich contextual information. For the application scenario of small object detection at construction sites, this paper proposes the BE-AFFM as a replacement for the SPPF module.

**Figure 3:**
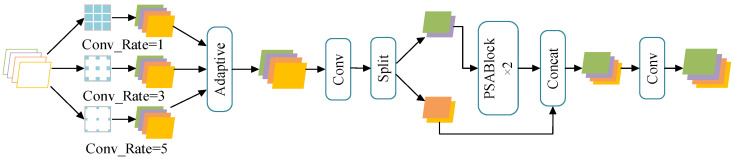
BE-AFFM.

The BE-AFFM employs dilated convolution operations, allowing adjustment of the receptive field size through the dilation rate parameter. Compared to the pooling operations in the SPPF layer, this offers greater flexibility and captures a broader range of contextual information. As illustrated in Figure 3, the module first applies dilated convolution to the data using three 3 × 3 convolution kernels with a stride of 1, set to dilation rates of 1, 3, and 5, respectively. The data undergoes adaptive feature fusion following the dilated convolution. Subsequently, a splitting operation divides the data into two parts along the channel dimension. One part undergoes processing through n layers of PSABlock before output, which is then concatenated along the channel dimension with the other part of the data. Finally, a convolution operation restores the data to its original input dimensions. The value of n is determined by detection requirements; in this study, n is set to 2.

**Figure 4:**
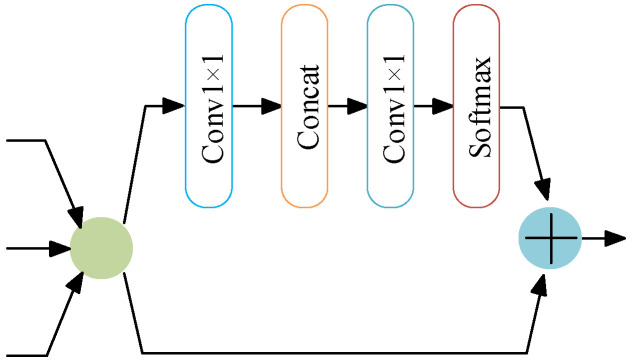
Adaptive Module.

The adaptive feature fusion framework adopted by the BE-AFFM is shown in Figure 4. After dilated convolution, the feature maps are divided along the channel dimension. One branch undergoes convolution, concatenation, and normalization, and is then added back to the other branch to generate the module output. The dilated convolution operation triples the number of channels, allowing the model to capture a broader contextual range. Through adaptive feature fusion, redundant information is suppressed while salient features are emphasized, effectively reducing the number of channels and easing the computational load for subsequent layers.

**Figure 5:**
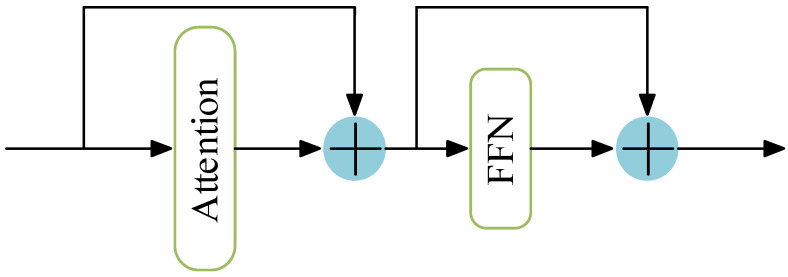
PSABlock Module.

The internal structure of the PSABlock, embedded within the BE-AFFM, is depicted in Figure 5. The PSABlock integrates a multi-head attention mechanism that strengthens the model’s ability to capture key features. The computation process is defined by Equation (5):

(5) $$\mathrm{Attention}\left(Q,K,V\right)=\mathrm{softmax}\left(\frac{QK^{T}}{\sqrt{d_{K}}}\right)V$$

where *Q* (Query) denotes the query matrix; *K* (Key) represents the key matrix; *V* (Value) signifies the value matrix; indicates the key dimension, used to adjust numerical magnitude; Softmax normalizes the attention scores.

Compared with the pooling operation used in SPPF, the BE-AFFM employs dilated convolutions to more flexibly capture multi-scale contextual information. This design achieves efficient multi-scale feature fusion while the integrated attention mechanism highlights information from key regions. Additionally, the Split operation enables multi-path feature extraction, providing richer gradient information and enhancing the network’s overall representational capacity.

The mathematical expression for the BE-AFFM is as follows:

(6) $$X_{1}=\mathrm{Adaptive}\left(\mathrm{Concat}\left({\mathrm{Conv}}_{r=1}\left(X\right),{\mathrm{Conv}}_{r=3}\left(X\right),{\mathrm{Conv}}_{r=5}\left(X\right)\right)\right)$$

(7) $$\mathrm{Split}\left(X_{2},X_{3}\right)=\mathrm{Split}\left({\mathrm{Conv}}_{1\times 1}\left(X_{1}\right)\right)$$

(8) $$F_{BE-AFFM}=\mathrm{Conv}\left({\mathrm{PSABlock}}^{\left(2\right)}\left(X_{2}\right),X_{3}\right)$$

where *X* denotes the input data, and $X_{1}$, $X_{2}$ and $X_{3}$ represent intermediate variables.

**Figure 6:**
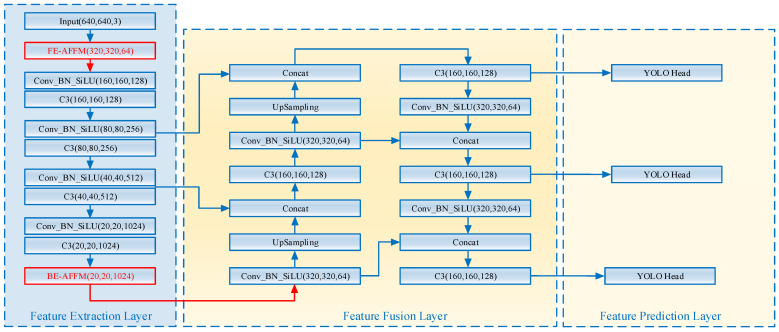
The Architecture of the DAF-YOLO Network.

The improved network architecture is illustrated in Figure 6. Introducing the FE-AFFM at the head of the YOLOv5s backbone enhances the model’s data mining capabilities at source, providing richer information for subsequent feature extraction modules. Integrating the BE-AFFM at the tail of the backbone enables more flexible feature consolidation.

## 4. Experimental Results and Analysis

### 4.1. Experimental Environment and Dataset Preparation

This paper uses a cloud server platform to conduct experiments on a self-made dataset. The specific experimental environment is listed in Table 1. Based on the reference data provided by YOLO, the training parameters are set as follows: the number of training rounds is set to 150, the experimental batch size is set to 16, the initial learning rate is set to 0.01, and the Adam optimizer is used to optimize the model parameters.

In Experiment 4.7, the environment used for training the public dataset is shown in Table 2. Training was conducted according to the parameters set in the paper [24,25], with an experimental batch size of 16, a training epoch size of 100, and an initial learning rate of 0.01.

**Figure 7:**
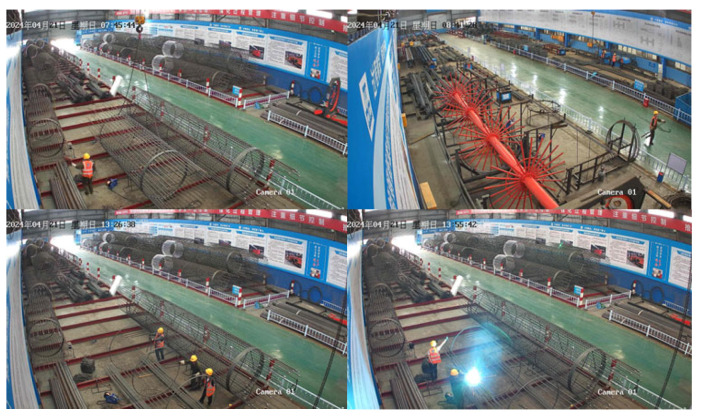
Sample images from the self-made dataset.

The datasets used in the experiments included both the self-made dataset and the publicly available dataset. The self-made dataset consisted of real photographs taken by cameras installed at a high point on the construction site. The construction site environment is complex, with significant differences in lighting at different times of day, and the activity area of the detected targets is wide, leading to substantial differences in the scale of the detected targets in different areas. The original dataset consisted of 1055 images. To increase data diversity and meet actual engineering needs, the images were appropriately processed, such as by horizontal flipping, resulting in a final dataset of 2110 images. LabelImg (version 1.8.6.) software was then used to annotate the images, creating a dataset containing 10,302 targets. Some sample images from this self-made dataset are shown in Figure 7.

**Figure 8:**
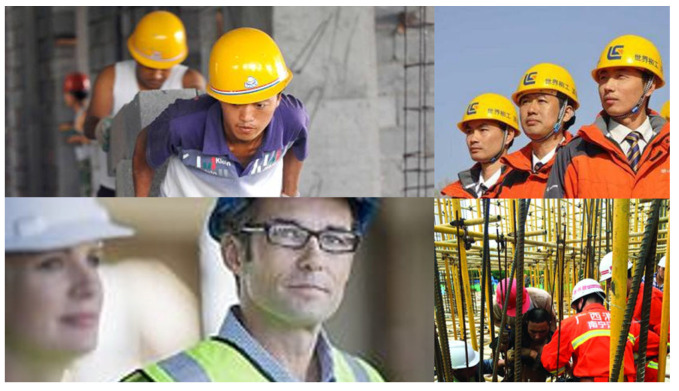
SHWD Sample.

To more objectively demonstrate the superiority of the improved algorithm, this study also used a publicly available dataset for testing. The selected Safety-Helmet-Wearing-Dataset is an open-source dataset used to detect whether workers are wearing safety helmets. This dataset contains 7581 images, including 9044 positive samples of workers wearing safety helmets and 111,514 negative samples of workers not wearing safety helmets. Some sample images from SHWD are shown in Figure 8.

Table 3 details the specific quantities of each target category within the custom dataset. In the table, ‘Hat’ denotes workers wearing safety helmets, ‘No_hat’ denotes workers not wearing safety helmets, ‘Vest’ denotes workers wearing high-visibility vests, and ‘No_vest’ denotes workers not wearing high-visibility vests.

### 4.2. Experimental Evaluation Metrics

This experiment employs metrics commonly used in object detection tasks—precision (*P*), recall (*R*), and mean average precision (*mAP*)—to evaluate model accuracy. These metrics are calculated according to the following formulae:

(9) $$P=\frac{TP}{TP+FP}$$

(10) $$R=\frac{TP}{TP+FN}$$

(11) $$mAP=\frac{1}{c}\sum_{i=1}^{c}P_{i}$$

where *TP* (True Positive) denotes correctly predicted instances belonging to the positive class; *FP* (False Positive) denotes false alarms where instances actually belonging to the negative class are classified as positive; *FN* (False Negative) denotes missed detections where instances actually belonging to the positive class are classified as negative; *C* represents the number of target object classes; denotes the precision for detecting each unsafe behavior.

### 4.3. Ablation Studies

This study introduces two improved methods designed to enhance the detection of small-scale unsafe behaviors at construction sites. To assess the effectiveness of each improvement, ablation experiments were carried out on the self-constructed dataset under identical experimental settings. The results are summarized in Table 4.

In the table, the symbol “√” indicates that the module was applied, while “–” denotes that it was not. As shown in Table 2, adding the FE-AFFM improved the model accuracy by 3.7%. When only the BE-AFFM was integrated, the performance gains were smaller; however, when both modules were used together, all evaluation metrics showed noticeable improvement. This is mainly because the FE-AFFM strengthens feature extraction at the front end, providing richer feature information to the BE-AFFM at the end of the backbone network, which in turn performs more efficient feature fusion.

Overall, both enhancements contribute positively to the network’s performance. Compared with the baseline algorithm, the improved model achieves an increase of 3.6% in mAP@0.5 and a 9.5% improvement in mAP@0.5:0.95, indicating a substantial boost in detection accuracy. These results confirm that the proposed algorithm performs well in complex construction site environments and effectively improves small-target detection.

### 4.4. Algorithm Comparison

To comprehensively evaluate the performance of the improved YOLOv5s model, several representative lightweight detection algorithms were selected for comparison under identical experimental settings. All models were trained and tested on the self-constructed dataset of unsafe behaviors at construction sites, and the results for key performance metrics are summarized in Table 5.

As can be seen from the table, among the tested models, the YOLOv3-Tiny model achieved an mAP@0.5 of 86.9% and a precision of 94.9%, but its recall remained relatively low at only 81.8%. This indicates that in complex architectural scenes, this model often misses a considerable number of targets.

In contrast, the YOLOv4 algorithm’s overall performance was weaker, with an mAP@0.5 of 83.6%, a precision of 86.8%, and a recall of only 78.5%. The low recall rate suggests that this algorithm performs poorly in small target detection scenarios, easily leading to missed detections of small targets in complex architectural environments.

Compared to the above algorithms, YOLOv5s performed significantly better, with an mAP@0.5 of 92.1%, a precision of 97.6%, and a recall of 87.8%. This algorithm achieved a good balance between precision and recall.

This paper also selected YOLOv10n and YOLOv12n for reference. Experiments showed that although these two algorithms were released more recently, their detection accuracy on the self-made dataset was actually lower than that of YOLOv5s. This may be because their model architecture is more inclined to optimize for general datasets, resulting in poor generalization ability on specific scenario data. Meanwhile, to verify the effectiveness of the proposed module, the FE-AFFM and BE-AFFM were introduced into the YOLOv12n network structure. The improved DAF-YOLOv12n showed significant improvements in various metrics, with mAP@0.5 increasing by 5.3% and recall increasing to 91.5%.

The proposed DAF-YOLO algorithm achieved the best results on all evaluation metrics. The improved algorithm achieved an mAP@0.5 of 95.7%, a 3.6 percentage point improvement over the baseline model; precision reached 98.4%; and recall significantly improved to 92.8%, a 5 percentage point improvement over the baseline model. This demonstrates that DAF-YOLO is highly effective in small target detection and significantly reduces false negatives.

The comparative experimental results of various algorithms clearly confirm the superiority of the improved algorithm. DAF-YOLO achieves higher recall while maintaining excellent precision, indicating that it can more comprehensively identify unsafe behaviors in complex construction scenarios without introducing excessive false positives. This algorithm lays a solid technical foundation for its application in practical construction safety monitoring.

### 4.5. Visualization of Model Detection Results

In object detection tasks, researchers often choose mean average precision (*mAP*) as a key metric for evaluating model performance. During model training, the dataset typically contains multiple detection classes, each generating a corresponding precision-recall curve. The area under the curve represents the mean precision (AP) for that class. *mAP* is obtained by calculating the arithmetic mean of the AP values for all classes, reflecting the balance between precision and recall. A well-performing model will have its curve covering a large area near the upper right corner, maintaining high precision even with high recall.

**Figure 9:**
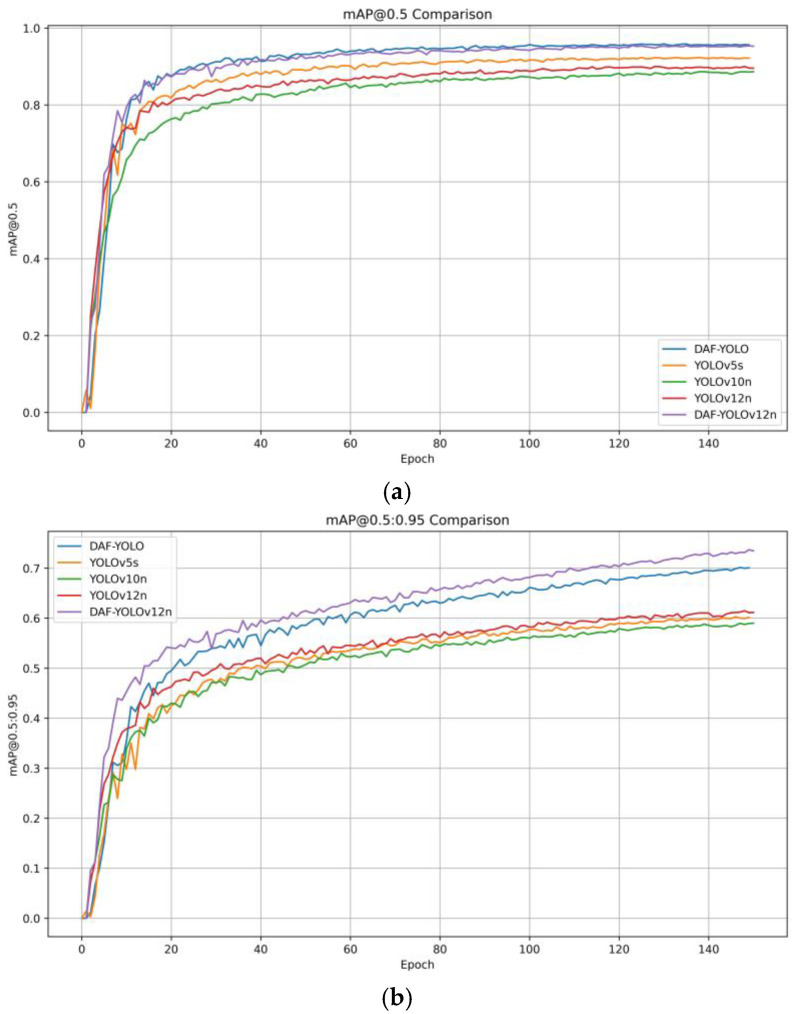
Mean Average Precision Comparison. (**a**) mAP@0.5 Comparison; (**b**) mAP@05:0.95 Comparison.

To visually demonstrate the detection performance of the improved algorithm, we conducted comparative experiments with several typical algorithms and the improved model. We also presented the *mAP* curves for each algorithm. Figure 9 shows the experimental results and the changes in training parameters with the number of training epochs.

As shown in the figure, in the initial training phase of the model, the advantages of DAF-YOLO and DAF-YOLOv12n are not obvious. In the first ten rounds or so, YOLOv12n even maintains a lead over DAF-YOLO. After the fifteenth round of training, the convergence speed of the improved algorithm increases rapidly, gradually widening the gap with YOLOv5s, YOLOv10n and YOLOv12n. This gap is more obvious in Figure 9b.

**Figure 10:**
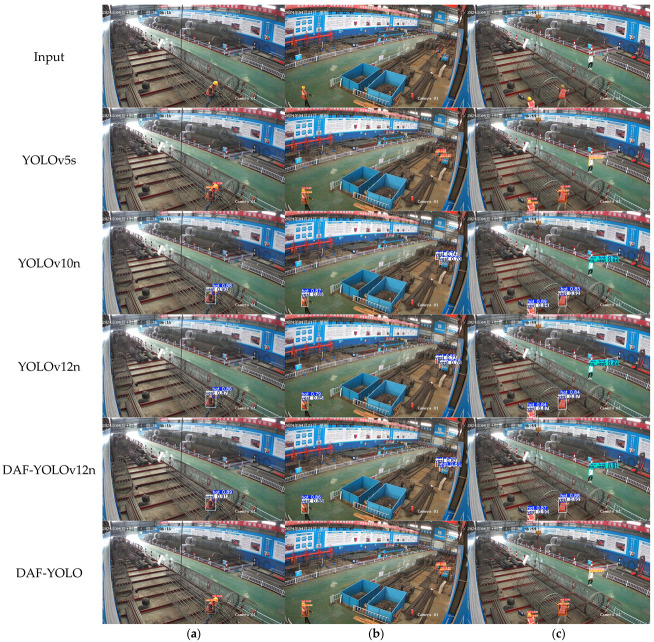
Comparison of actual detection results. (**a**–**c**) represent different picture examples.

Images were randomly selected from the test set to test the target detection performance of the original and improved models, as shown in Figure 10. From Figure 10a, it is clear that when the construction worker is close to the camera, the performance of the various algorithms listed is similar, indicating that the YOLO algorithm has superior detection performance for normal-scale targets. However, when the construction worker is far from the camera, the pixel proportion of the safety helmet in the image decreases sharply, as shown in Figure 10b. YOLOv5s’ detection accuracy for the safety helmet on the right side of the image is only about 50%, while YOLOv10n and YOLOv12n’s detection accuracy for this target is slightly better than YOLOv5s, but only maintains a level of around 60%. The improved DAF-YOLOv12n and DAF-YOLO have a more sensitive detection capability for small targets in the image. The former maintains a detection accuracy of around 80% for the safety helmet, while the latter’s detection accuracy is close to 90%, significantly outperforming other algorithms. Furthermore, it is clearly shown in Figure 10c that the improved algorithm significantly enhances its ability to detect construction workers not wearing safety helmets and reflective vests, improving by 7% and 5%, respectively, compared to the baseline model. This indicates that the improved model has the ability to accurately detect small targets in complex construction site backgrounds.

### 4.6. Heatmap Analysis

**Figure 11:**
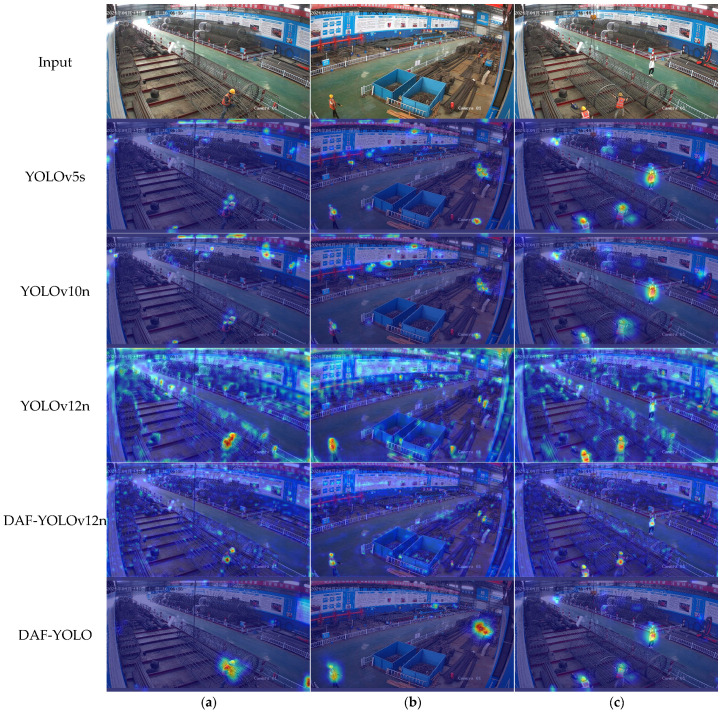
Heatmap Visualization. (**a**–**c**) represent different picture examples.

Heatmap analysis is a data visualization technique that uses varying shades of color to intuitively display the distribution, density, and intensity of data across a spatial or graphical plane. It translates numerical values into a color spectrum, where warm colors typically denote high values or dense regions, while cool colors represent low values or sparse areas. To more clearly observe the degree of attention paid to different image regions during object detection tasks by the improved model versus the baseline model, heatmap analysis was conducted on images from the test set, as shown in Figure 11.

As clearly seen in the images, various materials are haphazardly piled up at the construction site, and the posters on the walls are cluttered and diverse. This easily generates negative feature information during image detection, causing the detection model to pay unnecessary attention. Compared to YOLOv5s, the improved DAF-YOLO significantly enhances its resistance to environmental interference, noticeably reducing its focus on non-critical areas. YOLOv12n performs the worst among the listed algorithms, especially in Figure 11a, where traffic warning posts at the construction site, due to their similar color and shape to reflective vests, attract excessive attention from YOLOv12. The improved DAF-YOLOv12n effectively mitigates this issue. Research indicates that the module proposed in this paper can extract and fuse feature information at depth, greatly reducing the probability of identifying erroneous features.

**Figure 12:**
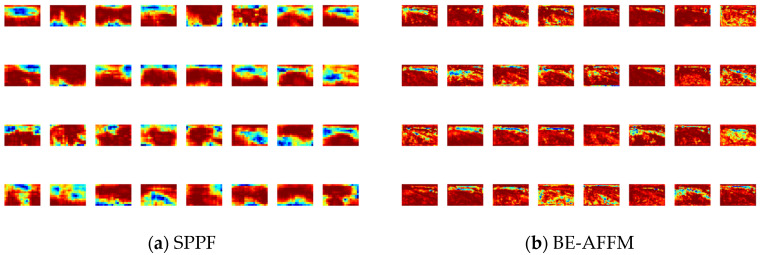
Feature map visualization.

This paper compares the SPPF module in the baseline model with the improved BE-AFFM, visualizes the feature maps output by the two models, and analyzes their respective performance. As can be seen from Figure 12a, the feature map processed by SPPF has a smoother and more blurred region of interest, while the feature map processed by the BE-AFFM in Figure 12b has a clear sense of hierarchy, which greatly improves the nonlinear expressive ability of the model and enables more efficient feature fusion.

### 4.7. Algorithm Comparison on Public Datasets

This study compared several classic target detection algorithms on the publicly available Safety Helmet Wearing Dataset (SHWD), including Faster R-CNN, RFBNet, SSD, various YOLO series models, and the proposed DAF-YOLO algorithm. The experimental results are summarized in Table 6.

As shown in the table, the traditional two-stage detection algorithm Faster R-CNN achieved an mAP@0.5 of 87.4%, indicating relatively high detection accuracy. Among the single-stage algorithms, RFBNet and SSD achieved mAP@0.5 values of 75.6% and 84.2%, respectively, indicating relatively low performance in accurately detecting small targets. This study selected YOLOv5, YOLOx, and YOLOv8 from the YOLO series for experiments, achieving mAP@0.5 scores of 85.7%, 88.3%, and 86.5%, respectively. This demonstrates that the YOLO series algorithms achieve a good balance between detection speed and accuracy, and their overall performance is superior to traditional methods.

Compared to the algorithms mentioned above, the proposed DAF-YOLO model consistently achieves the best performance across key metrics. Its mAP@0.5 reaches 93.8%, and mAP@0.5–0.95 reaches 60.5%, with a precision of 92.4% and a recall of 89.6%. The improved precision and recall demonstrate that DAF-YOLO not only detects targets more accurately but also reduces missed detections of small targets. These results confirm that the dual adaptive feature fusion strategy effectively enhances feature representation and multi-scale detection capabilities, validating the superiority and practical applicability of DAF-YOLO on the SHWD.

## 5. Conclusions

To achieve efficient detection and identification of unsafe worker behaviors in complex construction site scenarios, this paper proposes an improved algorithm—DAF-YOLO—based on YOLOv5s. This algorithm performs dual feature enhancement optimizations on the original network structure: FE-AFFM are introduced at the beginning of the backbone network to enhance the module’s deep feature mining capabilities from the source; at the end of the backbone network, the original SPPF module is replaced with a BE-AFFM, achieving more efficient and flexible feature information fusion. Through this improvement, the network can achieve more accurate semantic representation in the multi-scale feature extraction and fusion stages, effectively solving the problem of small target detection in complex construction site environments, thereby significantly improving the detection performance of unsafe behaviors.

To verify the effectiveness of the proposed algorithm, comparative experiments were conducted on a self-made dataset of unsafe worker behaviors on construction sites and a publicly available dataset of safety helmet wearing. Experimental results show that DAF-YOLO outperforms the baseline model YOLOv5s in both detection accuracy and recall. On a self-made dataset, the improved algorithm’s mAP@0.5 increased by 3.6%, especially the mAP@0.5–0.95 range, which increased by 9.5%. Furthermore, DAF-YOLO also outperforms other algorithms on publicly available datasets. The experiments demonstrate that DAF-YOLO can achieve higher detection stability and robustness in complex construction site environments, fully proving the superiority of the improved algorithm.

Despite achieving relatively ideal experimental results, there is still room for improvement. Due to drastic changes in lighting and frequent occlusion in construction site scenarios, the model’s detection accuracy still decreases under some extreme conditions. Future research will focus on improving dataset quality, optimizing network structure, and lightweight model design to further reduce computational and storage overhead. Simultaneously, efforts will be made to integrate behavior recognition and tracking modules into the improved algorithm to build a more intelligent and efficient construction site safety monitoring system.

## Figures and Tables

**Table 1:** Experimental configuration and platform used for self-made dataset.

Component	Parameters
CPU	15vCPU Intel(R) Xeno(R) Platinum 8474C
RAM	80 G
GPU	RTX 4090D (24 GB)
Programing Language	Python3.8
Deeplearning Framework	PyTorch 1.10.1
CUDA	11.3

**Table 2:** Experimental configuration and platform used for the SHWD.

Component	Parameters
GPU	RTX 3090 (24 GB)
IDE	Pycharm
Programing Language	Python3.8.15
Deeplearning Framework	PyTorch 1.13.1
CUDA	11.6

**Table 3:** Object classifications and quantities in the custom dataset.

Defect Type	Training Set	Validation Set	Total
Hat	3200	812	4012
No_hat	978	216	1194
Vest	2670	664	3334
No_vest	1425	337	1762

**Table 4:** Ablation Experiments.

FE-AFFM	BE-AFFM	mAP@0.5 (%)	mAP@0.5–0.95 (%)	*P* (%)	*R* (%)
–	–	92.1	60.5	97.6	87.8
√	–	95.8	68.3	97.7	92.9
–	√	92.4	61.7	96.7	89.2
√	√	95.7	70.0	98.4	92.8

**Table 5:** Algorithm Comparison on Custom Dataset.

Model	mAP@0.5 (%)	*P* (%)	*R* (%)
YOLOv3Tiny	86.9	94.9	81.8
YOLOv4	83.6	86.8	78.5
YOLOv5s	92.1	97.6	87.8
YOLOv10n	88.6	95.4	81.4
YOLOv12n	90.0	98.0	83.9
DAF-YOLOv12n	95.3	97.5	91.5
DAF-YOLO	95.7	98.4	92.8

**Table 6:** Algorithm Comparison under SHWD.

Model	mAP@0.5 (%)	mAP@0.5–0.95 (%)	*P* (%)	*R* (%)
Faster R-CNN [24]	87.4	54.1	89.5	80.5
RFBNet [24]	75.6	41.6	82.6	73.4
YOLOv5 [24]	85.7	55.4	88.4	78.6
YOLOX [24]	88.3	54.9	91.0	81.7
YOLOv8 [24]	86.5	54.2	89.2	79.5
SSD [25]	84.2	51.6	88.3	76.5
DAF-YOLO	93.8	60.5	92.4	89.6

## Data Availability

The Safety Helmet Wearing Dataset (SHWD) used in this study is publicly available at the following URL: https://github.com/njvisionpower/Safety-Helmet-Wearing-Dataset (accessed on 1 August 2025).
